# Structure and Function of Ocular Proteoglycans: Essential Proteins for Vision

**DOI:** 10.3390/ijms27041943

**Published:** 2026-02-18

**Authors:** James Melrose

**Affiliations:** 1 Raymond Purves Bone and Joint Laboratory, Kolling Institute of Medical Research, The University of Sydney at Royal North Shore Hospital, Northern Sydney Local Health District, St. Leonards, Sydney, NSW 2065, Australia; james.melrose@sydney.edu.au; 2 Graduate School of Biomedical Engineering, University of New South Wales, Sydney, NSW 2052, Australia

**Keywords:** hyaluronan, SLRPs, lecticans, synapse, pikachurin, eyes-shut, neurexin, vision

## Abstract

This narrative review outlines the structure and essential functions of ocular proteoglycans (PGs) in visual processing as documented in the extensive literature on this subject matter. The eye, as one of the most complex sensory organs, relies on the coordinated activity of various tissues and cell types, with PGs playing a central role in facilitating communication and maintaining tissue function. These molecules stabilise ocular tissues; for example, SPACRCAN (IMPG2) and hyaluronan aggregates in the interphotoreceptor matrix protect photoreceptors from oxidative stress. Specialised heparan sulfate PGs, such as pikachurin, eyes-shut, and the neurexin family, stabilise synapses and ensure synaptic specificity and plasticity. Pikachurin is particularly important for the rapid transmission of visual signals at the bipolar ribbon synapse. A diverse array of chondroitin sulfate (aggrecan, versican, neurocan, brevican, phosphacan, NG2), keratan sulfate (SV2), and heparan sulfate (perlecan, agrin, collagen XVIII) PGs are differentially expressed in ocular tissues, contributing to tissue stability and homeostasis. In the cornea, sclera, and choroid, small leucine-rich repeat PGs (SLRPs) maintain three-dimensional structure, corneal transparency, and tissue function through interactions with cytokines and growth factors. The vitreous humour contains opticin and nyctalopin, which support the nutrition of avascular regions and facilitate bipolar ribbon synapse signalling. Ultimately, the effectiveness of the eye as a visual organ depends significantly on the functional roles of its constituent PGs.

## 1. Introduction

The eye is a highly complex sensory organ, comprising multiple specialised tissues and diverse cell types that work together to capture visual information and transmit it as electrical signals through the retinal neural network to the optic nerve and ultimately the brain for interpretation [1,2]. Proteoglycans (PGs) play essential roles in the structure and function of these ocular tissues. This study aims to provide an overview of the key PGs present in the eye and their functional significance within its various components. An understanding of the unique anatomical and functional features of the eye is fundamental to appreciating the importance of these molecules.

## 2. The Sclera

**Figure 1:**
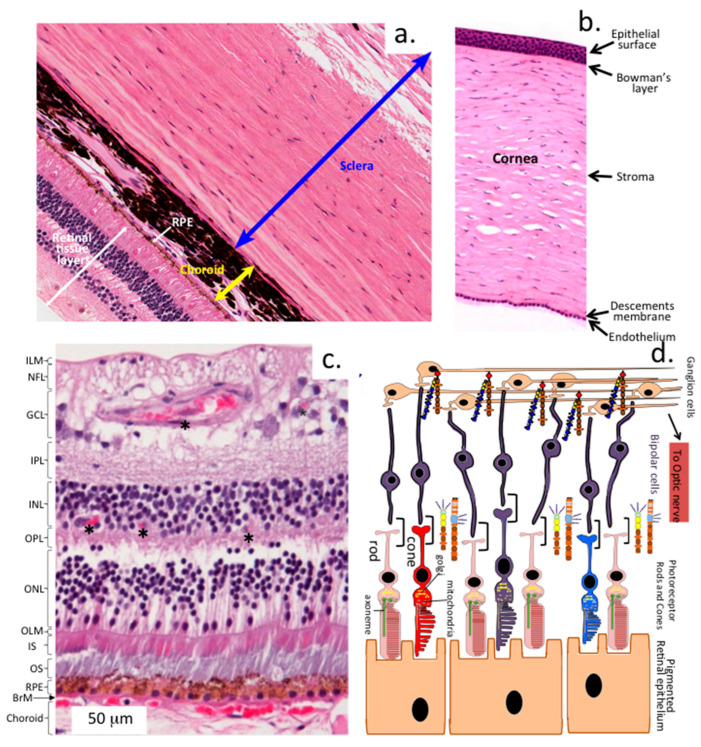
Histological sections of the sclera (**a**), cornea (**b**), and retina (**c**). The sclera is a tough collagen rich tissue that merges with the choroid which is a vascular tissue which supplies nutrition to the retina pigmented epithelium (RPE) and the various layers of the retina. The cornea is also a collagen rich tissue containing a surface epithelium, Bowman’s layer, stroma, and an underlying endothelium and Descemets membrane. Ten layers have been identified in the retina. Light entering the eye passes through dense layers of neuronal cells and blood vessels (asterisks) before it is absorbed in the outer segments of the photoreceptors generating an electrical signal (phototransduction); this signal is transferred to bipolar neurons in the retinal neural network and eventually to ganglionic neurons, which is then transmitted to the brain via the optic nerve (**d**). Abbreviations: ILM, inner limiting membrane; NFL, nerve fibre layer; GCL, ganglion cell layer; IPL, inner plexiform layer; INL, inner nuclear layer; OPL, outer plexiform layer; ONL, outer nuclear layer; OLM, outer limiting membrane; IS, photoreceptor inner segments; OS, photoreceptor outer segments; RPE, retinal pigment epithelium; BrM, Bruch’s membrane; Choroid, anterior part of the choroid. A schematic is shown of the RPE communicating with the photoreceptors, bipolar neurons, and ganglionic neurons which transmit signals generated in the photoreceptors to the optic nerve and hence to the brain for visual processing. Several specialised HSPGs, pikachurin, eyes-shut, and neurexin stabilise synaptic interactions in this neuronal mosaic. Pikachurin and eyes-shut stabilise the bipolar neuron ribbon synapse. Neurexin also stabilises synapses through interactions with a large range of binding proteins that provide specificity to synaptic interactions and synaptic plasticity. A CS–proteoglycan, SPACRCAN, forms complexes with hyaluronan which stabilise the interphotoreceptor matrix (IPM), protect the photoreceptors from oxidative damage, and provide interconnectivity with the RPE. These are not shown in this figure to avoid overcrowding of figure. Images reproduced from [24] (open access).

The sclera is a dense, collagen-rich tissue that encircles the eye and merges anteriorly with the cornea [3,4]. It consists of three layers: fibrous, vascular, and sensory (Figure 1). The outermost layer, the sclera proper, is a tough, white, fibrous tissue that maintains ocular shape and protects internal structures. Extraocular muscles attach to the sclera, enabling eye movement. Microscopically, the sclera displays concentric layers, including Tenon’s capsule, episclera, stroma, and lamina fusca, which transitions into the underlying choroid [3,5]. The stroma contains interlacing bundles of collagen types I, III, V, and VI, with elastic fibres abundant in the lamina fusca [6].

The extracellular matrix (ECM) of the sclera contains small leucine-rich repeat PGs (SLRPs) such as decorin, biglycan, keratocan, lumican, fibromodulin, and prolargin (PRELP), as well as large CSPGs like versican and aggrecan [7]. Decorin is closely associated with collagen fibrils and is involved in collagen fibrillogenesis [8], while FMOD and LUM regulate the formation of large and small collagen fibres, respectively. PRELP serves as an anchoring protein for other ECM components [9]. PGs in the sclera contribute to matrix stabilisation, hydration, solute diffusion, and fluid movement.

Proteomic studies have identified versican, aggrecan, decorin, biglycan, lumican, fibromodulin, keratocan, prolargin, and mimecan as key components of the scleral proteome [10,11,12,13,14,15]. Notably, increased aggrecan synthesis and accumulation in the posterior ECM of the sclera has been observed in myopic eyes [14,16], leading to alterations in the three-dimensional ECM structure and ocular enlargement. 

### The Choroid and Bruch’s Membrane

The choroid is a vascular layer supplying nutrients to the outer retina [17] and plays a role in lymphatic drainage and temperature regulation [18]. Its smooth muscle cells adjust choroidal thickness, affecting eye focus and potentially contributing to myopia. The choriocapillaris provides the main blood supply to the outer retina, while Bruch’s membrane (BrM) acts as a barrier [19,20,21], merging several basement membranes and containing PGs, such as perlecan, agrin, and collagen XVIII; these provide ECM stabilisation and regulate nutrient flow to the retinal pigmented epithelium (RPE) [22]. Ageing can lead to calcification of BrM [22,23], reduced HS content, and increased PG degradation, contributing to diseases like retinitis pigmentosa and amyotropic macular degeneration (AMD) [22]. Loss of HS chains in perlecan and collagen XVIII impairs BrM’s filtration and signalling functions, increasing disease risk.

## 3. The Cornea

**Figure 2:**
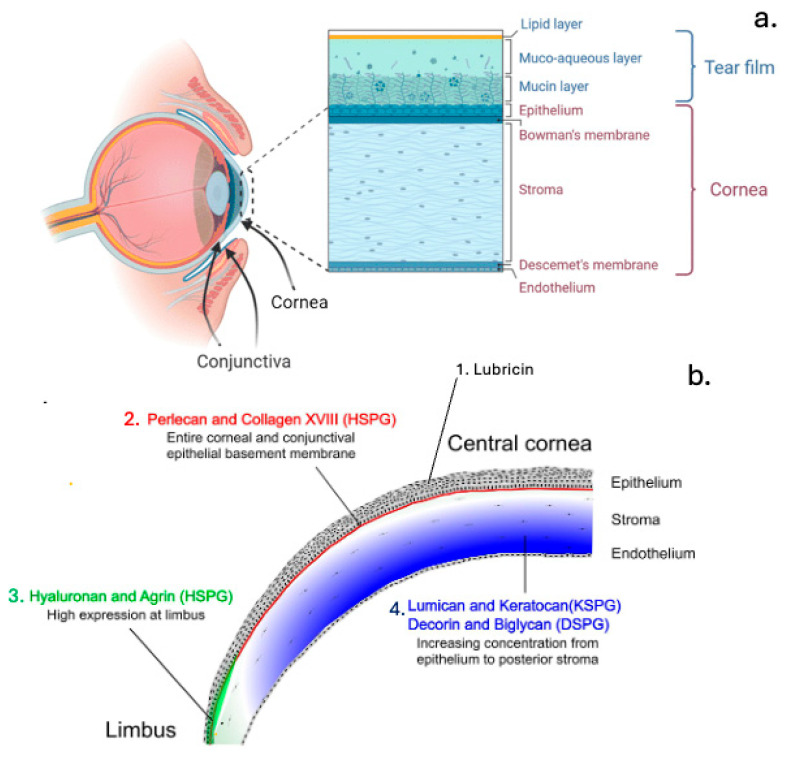
Schematic diagram of the cornea and conjunctiva organisation (**a**), and a lubricin diagram of the boundary lubricant proteoglycan in the cornea and the regionalised localisations of a number of other PGs in the cornea (**b**). Figure reproduced from [37] (open access).

The cornea, protruding through the sclera, contains highly organised collagen fibres for optical transparency [25] (Figure 2). SLRPs, including decorin, biglycan, lumican, keratocan, and fibromodulin bind collagen fibrils, help regulate fibrillogenesis as well as maintain corneal clarity and wound healing [26,27]. Lubricin (proteoglycan 4) acts as a surface lubricant, removing debris and reducing inflammation [28], but can be degraded in conditions like Sjögren’s syndrome [29] and dry eye syndrome [30]. The cornea’s layered structure and glycocalyx, rich in mucins and PGs, provide both refractive and protective functions [26,31,32,33]. Lumican is especially important for collagen organisation and optical clarity [34]. The cornea is also densely innervated, supporting debris clearance, epithelial health, and wound healing.

## 4. Biomechanical Roles of Ocular PGs

Ocular SLRPs, including decorin, biglycan, lumican, and keratocan, are integral functional components of the ECM of both the cornea and sclera. Although these PGs control collagen structure in each tissue, the way they affect biomechanics varies in the cornea and sclera, which is shown in the differences in tissue biomechanical properties [35].

### 4.1. Sclera

The sclera is a strong collagenous support tissue and its biomechnical properties have been compared with those of tendons. Decorin, biglycan, and aggrecan modulate the sclera viscoelastic properties, maintaining ocular shape under fluctuating intraocular pressure. PGs occupy interfibrillar spaces, facilitating stress transfer between collagen fibrils and ensure proper fibril spacing. This confers structural cohesion and resistance to elastic creep. Reduced PG-associated GAGs, as seen in myopia [36], increases scleral creep and weakens this tissue. The negative charge of GAG chains supports hydration and viscoelasticity. Scleral PG concentration rises until the fourth decade of life, enhancing stiffness, while after age 40, decreased decorin and biglycan levels may alter tissue compliance.

### 4.2. Cornea

Lumican, keratocan, and decorin are critical for forming thin, regularly spaced collagen fibrils, which underpin corneal transparency [37]. The highly charged KS chains of keratocan and lumican maintain optimal hydration, essential for stromal collagen architecture. Corneal GAGs contribute to tensile strength and mechanical integrity [36]. Decorin and lumican are also involved in wound healing, where they suppress myofibroblast activation and limit fibrosis, thereby preserving corneal transparency. Decorin and lumican are key regulators in tissue repair through the control of cytokine, with growth factor activities, such as structural ECM organisation and cell signalling during wound healing, are critical in the modulation of collagen fibrillogenesis, reducing scar formation and managing inflammation [26,38].

### 4.3. Biomechanical Studies Reveal Functional Changes in Corneal and Scleral Tissues in Disease

Biomechanical changes in the cornea and sclera occur in eye diseases like glaucoma, high myopia, and diabetes-related vision problems [39]. The sclera, mainly a support structure, also affects vision through its biomechanical properties [4]. The cornea is viscoelastic and anisotropic, with a stiffer anterior stroma due to higher collagen density. In keratoconus, corneal elasticity drops by up to 36%. Cross-linking treatments can increase corneal stiffness and slow disease progression, sometimes preventing the need for corneal transplants [40,41,42,43]. The sclera is nonlinear, viscoelastic, and anisotropic; its posterior regions are more extensible, and stiffness rises with age. High myopia causes posterior scleral thinning and weakening, while glaucoma makes softer scleral tissue more vulnerable to intraocular pressure, impacting the optic nerve. Increasing collagen cross-linking with agents like genipin or lysyl oxidase may help reverse or prevent these pathological changes [40,41,42,43].

## 5. The Iris and Lens

The iris is a diaphragm around the cornea, controlling pupil size and light entry. The lens absorbs, focuses, and directs light to the retina, with its shape regulated by peripheral muscles for optimal focus. The lens has a unique cellular structure and protein composition. Several PGs including syndecan (SDC) 1–4, glypican (GPC) 1–6, perlecan, collagen XVIII, and agrin are present in lens tissues. SDC and GPC are highly expressed during early lens development, while perlecan, collagen XVIII, and agrin become more prominent postnatally. HSPGs regulate growth factor signalling and drive morphogenetic changes in the lens. 

## 6. The Retina

**Figure 3:**
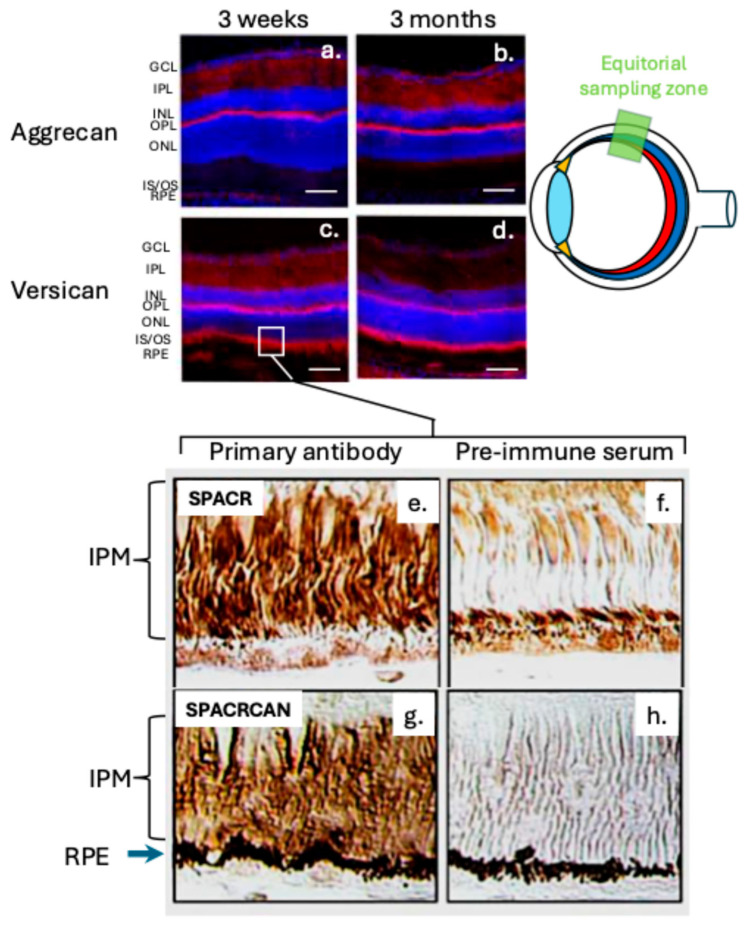
Retinal expression patterns of aggrecan and versican in wildtype mice (**a**–**d**). Confocal maximum projection images of the superior retina in the equatorial region. Aggrecan is restricted mainly to the GCL, IPL and OPL. Versican have a more widespread distribution through most retinal layers. Abbreviations: ONL, outer nuclear layer; OPL, outer plexiform layer; INL, inner nuclear layer; IPL, inner plexiform layer; GCL, ganglion cell layer; IS/OS, inner and outer segments of photoreceptors. Nuclei are counter-stained blue with DAPI-blue; CSPGs are stained red. Scale bar, 100 µm. A segment of the photoreceptor IS/OS region is depicted showing the immunolocalization of SPACR and SPACRCAN in the IPM (**e**–**h**). Scale bar 50 μm.

**Figure 4:**
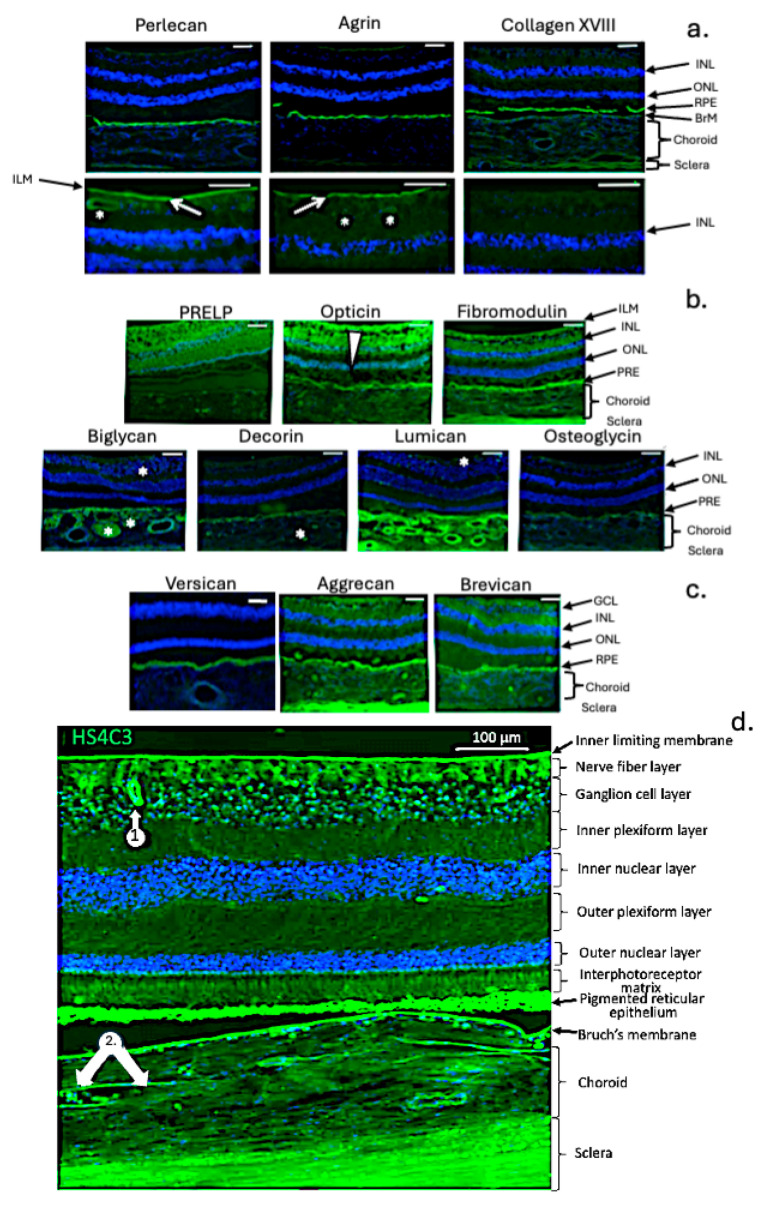
Immunolocalisation of retinal, scleral, and choroid PGs in the human eye. Basement membrane PGs (**a**), SLRPs (**b**), and CSPGs (**c**). The white arrows depict blood vessels. The basement membrane PGs perlecan, agrin, and collagen-XVIII are present in the internal limiting membrane, blood vessel walls, and Bruch’s membrane. Biglycan, decorin, fibromodulin, lumican, mimecan, opticin, and prolargin are differentially distributed in the retina, choroid, and sclera. Lumican is particularly prominent in the choroid. Versican, brevican and aggrecan are also detected. Versican is identified in Bruch’s membrane, while aggrecan and brevican are more widely distributed in the retina, choroid, and sclera. The white wedge symbol in Figure 4 shows the decreasing gradient of labelling for opticin from the internal limiting membrane through the neurosensory retina toward the choroid. The asterisks show the labelling of blood cells (e.g., leukocytes and erythrocytes) in the lumen of choroidal blood vessels and in the neurosensory retina. Fluorescent intensity in plate 4 (**d**) is enhanced to aid in the visualisation of the complex HS staining evident in retinal tissues. Scale bars are 100 μm in all plates. Images modified from [7] (open access).

The retina is a multilayered tissue with ten distinct layers and numerous cell types [44,45] (Figure 1c). Cone and rod photoreceptors transmit visual signals through a neural network of bipolar, horizontal, amacrine, Müller, and ganglion cells, ultimately sending information to the brain via the optic nerve. Aggrecan and versican are localised in the retina, with aggrecan mainly in the GCL, IPL, and OPL, and versican more widely distributed [46] (Figure 3a–d and Figure 4c). Neurocan and brevican are also present, while phosphacan is highly expressed during early development but decreases with age. Two CS PGs, SPACR (IMPG1) and SPACRCAN (IMPG2), are found in the interphotoreceptor matrix (IPM), where IMPG2 forms complexes with HA to protect photoreceptors from oxidative stress and support vision [47,48] (Figure 3e–h). NG2 proteoglycan (CSPG4) is expressed by oligodendrocyte precursor cells and pericytes in retinal blood vessels [49]. The HNK-1 carbohydrate, found on glycolipids, glycoproteins, and PGs like aggrecan, is involved in neural plasticity as a perineuronal net component [50]. Deficiency in HNK-1 impairs synaptic plasticity and spatial learning. Small leucine-rich repeat PGs (SLRPs) are abundant in the retina [51], modulating cell differentiation, adhesion, growth, repair, and signalling. Key SLRPs include biglycan, decorin, fibromodulin, lumican, PRELP, opticin, osteoglycin/mimecan, CHAD, Tsukushi, and nyctalopin [52].

### 6.1. The Macula

The macula is an oval region (~5 mm wide) in the retina responsible for central and colour vision, as well as high visual acuity. Its central area, the fovea centralis, contains densely packed cone photoreceptors for colour vision and sharp detail. Surrounding the fovea are rod photoreceptors, which are more numerous and enable black-and-white vision in low light [53].

### 6.2. The Photoreceptor

Photoreceptors are specialised cells in the retina [54] that convert light into electrical signals (phototransduction). Rods (~95% of photoreceptors; 100–125 million) function in dim light, while cones (~6 million) support colour vision in bright light [55]. The fovea contains only cones for maximum visual resolution [56], while rods and cones are mixed elsewhere in the retina. Light absorption triggers phototransduction, with signals relayed through the retinal network and optic nerve to the brain [57,58]. Photoreceptor cells are rich in lipids with membrane proteins forming stacks of discs containing light-absorbing pigments [56]. Rhodopsin (in rods) and opsin (in cones) both use 11-cis-retinal, which changes shape when exposed to light, activating G-proteins and initiating the visual signal [59]. Multiple cone pigments allow colour discrimination [60]. The RPE supports photoreceptors by supplying energy and removing spent cell fragments. The RPE adheres to BrM [61]; disruption of this can lead to neovascularisation and AMD, marked by drusen deposits and tissue bulging. Perlecan, agrin, and collagen XVIII in the RPE and BrM stabilise the ECM and anchor the RPE. Their HS chains interact with various proteins including complement factor H, which protects against AMD. Mutations in COL18A1 (collagen XVIII) can cause severe retinal disorders. Age-related increases in heparanase reduce HSPG function, contributing to AMD.

The interphotoreceptor matrix (IPM) between photoreceptors contains SPACRCAN (IMPG2), and SPACR (IMPG1). These form complexes with HA, protecting photoreceptors from oxidative stress and maintaining hydration. SPACR relies on SPACRCAN for proper incorporation into the IPM. The IPM also regulates nutrient and oxygen transport, growth factor sequestration, and the removal of photoreceptor debris. Disruption in these processes can lead to retinal degeneration. Photoreceptor tips are constantly renewed to sustain vision.

#### 6.2.1. Synaptic HSPGs

**Figure 5:**
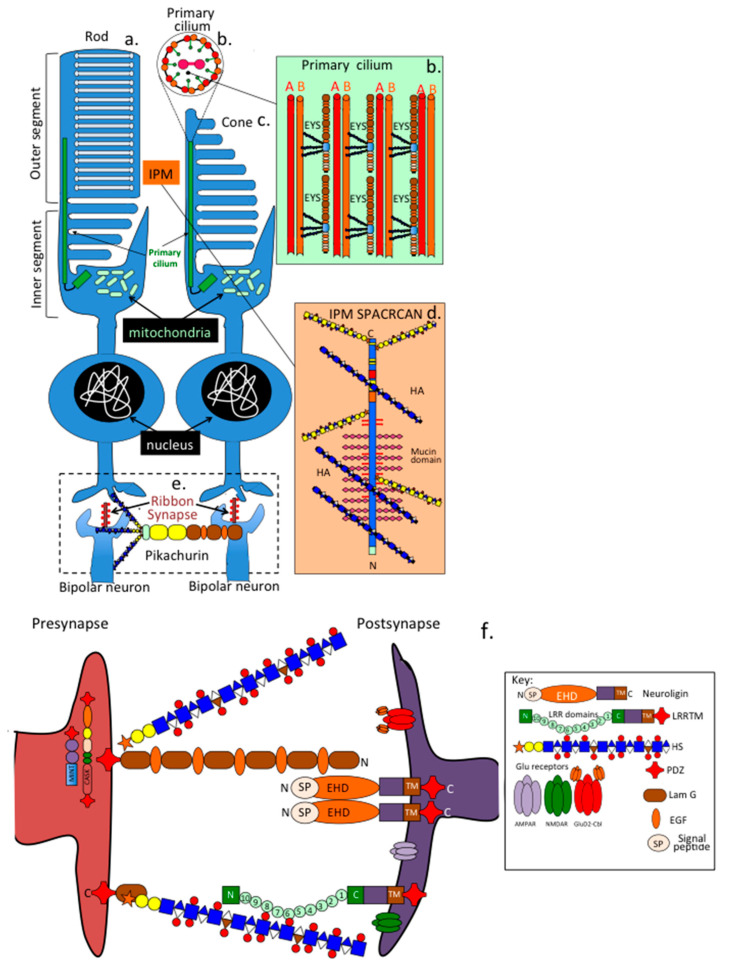
Schematics showing the stabilisation and protection of photoreceptors in the retina and the bipolar neuron ribbon synapse which interacts with photoreceptors and the other synapses in the retinal neural network. Stabilisation of the axonome primary cilium which stabilises the inner and outer segments of the rods (**a**) and cones (**c**) of the photoreceptors by eyes-shut (EYS). EYS supports the A and B microtubules in the 9 + 2 microtubular axonome arrangement in the primary cilium (**b**). SPACRAN-HA complexes stabilise the interphotoreceptor matrix (IPM) between the rods and cones (**d**). Pikachurin interacts with dystroglycan and dystrophin to stabilise the bipolar neuron ribbon synapse (**e**). Neurexin interacts with a large collection of binding proteins to provide synaptic stabilisation, specificity of interaction, and synaptic plasticity (**f**). Neuroligin and LRTMM (leucine-rich repeat transmembrane cell binding protein) are prominent neurexin ligands forming a dynamic synaptic cell adhesion network. Neurexin also interacts with glutamate receptors and CASK (calcium/calmodulin-dependent serine protein kinase) and MINT (CX-11 proteins) adaptor matrix proteins, providing synaptic stabilisation, specificity, and plasticity.

Several HSPGs have specialised functions in retinal neuronal networks. Eyes-shut stabilises the axoneme primary cilium connecting the inner and outer photoreceptor segments (Figure 5a–e). Pikachurin has specific interactions with dystrophin and dystroglycan in presynapses as well as the GPR179 orphan receptor on the post synaptic dendrite providing bipolar cell synapse stabilisation [62]. Neurexin also has roles in synaptic interactions with an extensive range of binding proteins providing synapse stabilisation, specificity and synaptic plasticity [63,64,65,66,67] (Figure 5f). The highly interactive HS chains of neurexin provide an increased level of ligands neurexin can interact with [66,68].

#### 6.2.2. The Bipolar Neuron Ribbon Synapse

**Figure 6:**
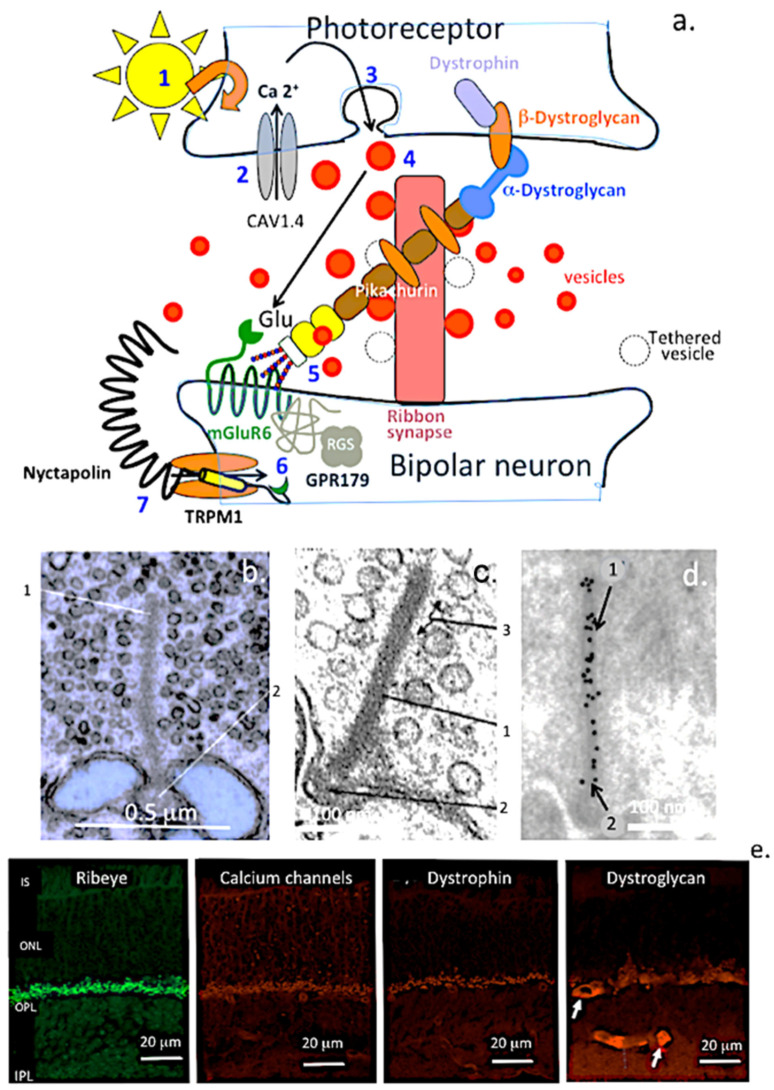
The bipolar neuron ribbon synapse. Schematic of the interactive components in the ribbon synapse (**a**). 1. Activation of light entry into photoreceptors results in; 2. an influx of Ca^2+^ into the presynaptic synapse and membrane polarisation resulting in; 3. a release of neuron synaptic vesicles containing neurotransmitters, primarily glutamate; 4. these vesicles attach to the central rod of the ribbon synapse; 5. glutamate released from these vesicles is taken up by mGluR6, metabromic glutamate receptor 6, and interacts with orphan receptor GPR179, which itself has roles in the polarisation of the postsynaptic neuronal dendrite and facilitates entry of metabolites. GPR179 is a G protein-coupled receptor that transmits extracellular messenger sensory stimuli to intracellular signalling pathways. The TRPM1, transient receptor potential melastatin cation channel M1, is coupled with mGluR6. Detection of glutamate by mGluR6 results in closing of the TRPM1 channel; and 6. light activation of photoreceptors resulting in glutamate release is halted and mGluR6 is deactivated. This results in opening of the TRPM1 channel, influx of sodium and calcium, and depolarization of the bipolar cell. Pikachurin interacts with dystroglycan dystrophin complex and the ribbon synapse components to provide synaptic stabilisation. Transmission electron images of the ribbon synapse show the central electron dense rod (1) attached to the synapse (2) surrounded by synaptic vesicles (3). (**b**,**c**). Immunogold labelling using a RIBEYE monoclonal antibody shows labelling along the entire rod of the ribbon synapse (**d**). Fluorescent immunolocalisation of RIBEYE, Ca channels, dystroglycan, and dystrophin shows these localised to the outer plexiform layer (OPL) of the mouse retina (**e**). Scale bars in (**e**) 20 μm. Dystroglycan also labels small blood vessels in the retina (arrow). Electron microphage images in b–d modified from [83,84,85] (open access). Fluorescent images in (**e**) modified from [69] (open access). Synaptic terminals of bipolar cells can contain 400,000–1,000,000 vesicles containing glutamate, a major neurotransmitter in visual processes [86].

The retina contains a specialised synapse called the ribbon synapse, found in both photoreceptors and bipolar cells [69,70,71] (Figure 6). Each cone cell has 10–40 ribbons (smaller than those in rods), and each bipolar neuron terminal can have up to 50 ribbons. The central structure of each ribbon is formed by the scaffolding protein RIBEYE [72]. Synaptic vesicles from photoreceptors attach to the ribbon’s central rod via 3–5 filaments [73]. Ribbon synapses are essential for transmitting signals from photoreceptors through the retinal network to ganglion cells, which then send visual information to the brain via the optic nerve.

#### 6.2.3. TRPM1 and the Bipolar Ribbon Synapse

Nyctalopin, a SLRP that interacts with TRPM1’s C-terminal domains in bipolar neurons, also involves mGluR6. Mutations in nyctalopin cause night blindness due to loss of rod function [74,75,76,77]. Pikachurin interacts with GPR179 and dystroglycan, stabilising the bipolar ribbon synapse [78,79]. Synaptic ribbons anchor vesicles in sensory neurons [80]. The N-terminal region of TRPM1 contains four melastatin homology domains (MHR) that help form the channel and transmit external signals. Its transmembrane domain has six helices (S1–S6), with S4 acting as a voltage sensor and the P-loop between S5 and S6 serving as the ion conduction pore [81]. The C-terminal region includes a conserved TRP helical domain and a coiled-coil domain, which enable complex formation with external ligands [82]. TRPM channels are vital for sensory systems. In mouse retinal ON-bipolar cells, TRPM1 is part of the cation channel regulated by mGluR6. Human TRPM1 mutations lead to congenital stationary night blindness, resulting in early childhood loss of rod function. Functional ON (depolarising) bipolar cells require the metabotropic glutamate receptor (mGluR6) and the TRPM1 cation channel for activity. Additionally, mGluR6 regulates the gating of TRPM1, and increased light intensity leads to depolarisation of ON-bipolar cells through glutamate release from presynaptic photoreceptors, with mGluR6 signalling to TRPM1 mediating synaptic transmission.

## 7. Ocular HSPGs in Health and Disease

HSPGs, including SDC1–4 and GPC1–6, are widely distributed in ocular tissues and play key roles in maintaining tissue homeostasis. Their diverse HS chains support functions such as cell adhesion, growth regulation, tissue development, anti-coagulation, angiogenesis, and defence against infection [87,88,89,90]. HS is present throughout the retina, RPE, BrM, choroid [91], basement membranes, and blood vessels [92], and is a component of perlecan, agrin, and collagen XVIII. The sulfation patterns of HS vary by location and are crucial for normal lens and eye development [93]. Disruption of HS structure can impair interactions with ECM components and growth factors, potentially leading to eye disorders. HSPGs show temporal and spatial variation in expression, influencing cellular proliferation and differentiation during eye development and maintenance.

### 7.1. Role of Heparan Sulfate in Age-Related Macular Degeneration

AMD occurs predominantly as an atrophic dry form and as a neovascular or “wet” form [94]. HS has roles in the pathogenesis of each of these conditions. Choroidal neovascularization (CNV) is a major cause of neovascular AMD where newly formed capillaries extend from the choroid to the BrM to gain access to the retina [95]. If this process is left unchecked, it will eventually lead to fibrosis beneath the macula, leading to a decrease in macular photoreceptor function through sensory retinal degeneration. CNV is regulated by various angiogenic agents, including growth factors, cytokines, and ECM components which interact with HSPGs [96]. HS promotes angiogenesis and ECM development and remodelling in both healthy and diseased states in the eye via interactions with cell receptors, HS binding, ECM proteins, angiogenic growth factors, inflammatory mediators, and anabolic growth factors such as PDGF, midkine, pleiotropin, FGF, VEGF, TNF-α, TGF-β, and IFN-γ [97].

#### HS Links the Complement Activation System and AMD

Studies have linked the complement system to the development of AMD, where the deposition of drusen in AMD activates the complement system [98]. AMD shares pathological and epidemiological similarities with the complement activation that occurs in atherosclerotic plaque formation [99]. Accumulation of drusen between the RPE and BrM is correlated with inflammation in ocular tissues and inadequate inactivation by the complement system [98,100]. Genetic variations in some complement proteins are strongly linked to an increased risk of developing AMD [101,102]. Complement factor H (CFH) promotes development of AMD [103,104]. CFH regulates the complement system and is critical in host cell and tissue protection [102]. A specific CFH gene polymorphism, Y402H, is associated with an increased risk of AMD development and can alter the protein’s specificity for HS [105], a major genetic risk factor for AMD [101]. The allotypic 402H CFH variant has decreased BrM and choroidal blood vessel binding sites compared to wildtype variant 402Y. This is where drusen deposits occur [106]. This reduced binding correlates with a significantly reduced HS content in perlecan and agrin in the BrM, as well as an age-related increase in heparanase activity [107].

### 7.2. Functional Roles for HSPGs in the RPE and Bruch’s Membrane

HSGs such as perlecan, agrin, and collagen XVIII stabilise the ECM and anchor the RPE to BrM [91]. Their diverse sulfate patterns enable interactions with a wide range of proteins, including ECM components, growth factors, cytokines, and complement system members [108,109,110]. HSPGs are localised in blood vessels and basement membranes, mediating connections between RPE cells and photoreceptors.

### 7.3. Roles for Collagen XVIII in Ocular Tissues

Collagen XVIII, a modular HSPG with three splice variants, is found in the RPE, BrM, choroid, and sclera [7]. It is essential for ECM stability and vision [111]. Mutations can cause Knobloch syndrome, leading to high myopia and retinal degeneration [112]. Its C-terminal endostatin domain may help control abnormal blood vessel growth in the retina [113].

### 7.4. Roles for Perlecan and Agrin in BrM and the RPE

Perlecan and agrin are present in BrM and the RPE. Agrin, in both soluble and transmembrane forms, is important for retinal development and synaptogenesis [114,115,116,117]. It binds growth factors like BMP2, BMP4, and TGF-β1, influencing tissue growth, regeneration, and immune regulation [118,119].

### 7.5. Roles for HSPGs in Neuro-Retinal Cells

Specific HSPGs are crucial for retinal synaptic assembly and function. Pikachurin interacts with α-dystroglycan to ensure synaptic specificity and forms connections between photoreceptors and bipolar cells [62,120,121,122,123]. Eyes-shut (EYS) stabilises the photoreceptor axoneme [124], while neurexin supports synaptic stability [125,126] and is involved in retinoid transport and rhodopsin maturation [127].

### 7.6. Aqueous Humour

Type IX collagen, a major component of the vitreous, exists in both PG and non-PG forms [128,129], with species-dependent CS chain variations. Opticin, a class III SLRP in the vitreous, binds retinal growth hormone, inhibits angiogenesis, and regulates collagen structure [130].

## 8. The Structure of Ocular PGs

**Figure 7:**
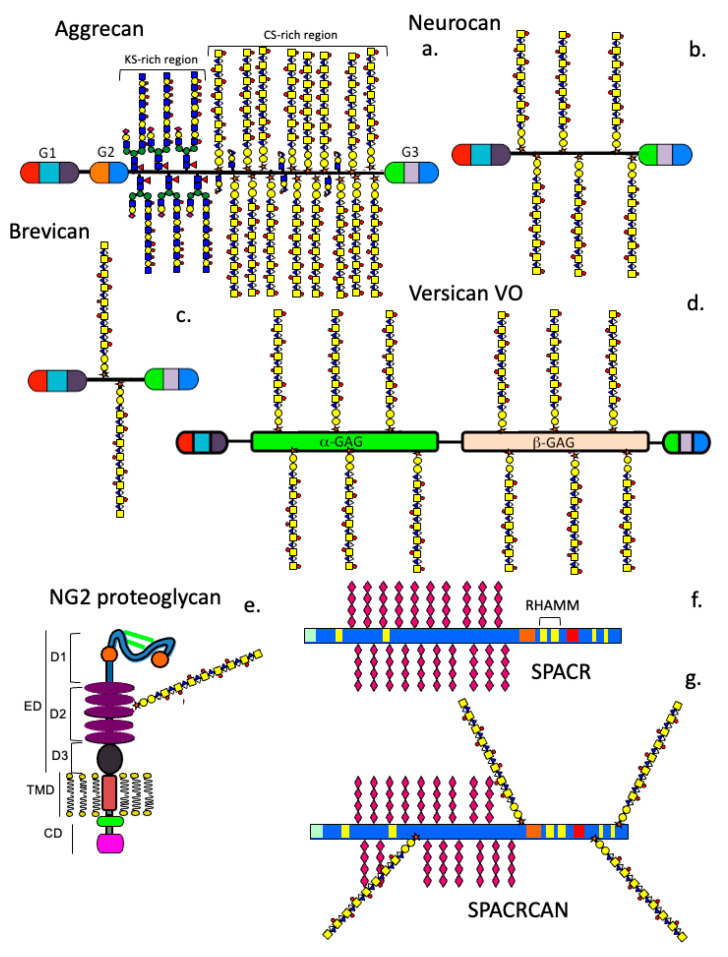

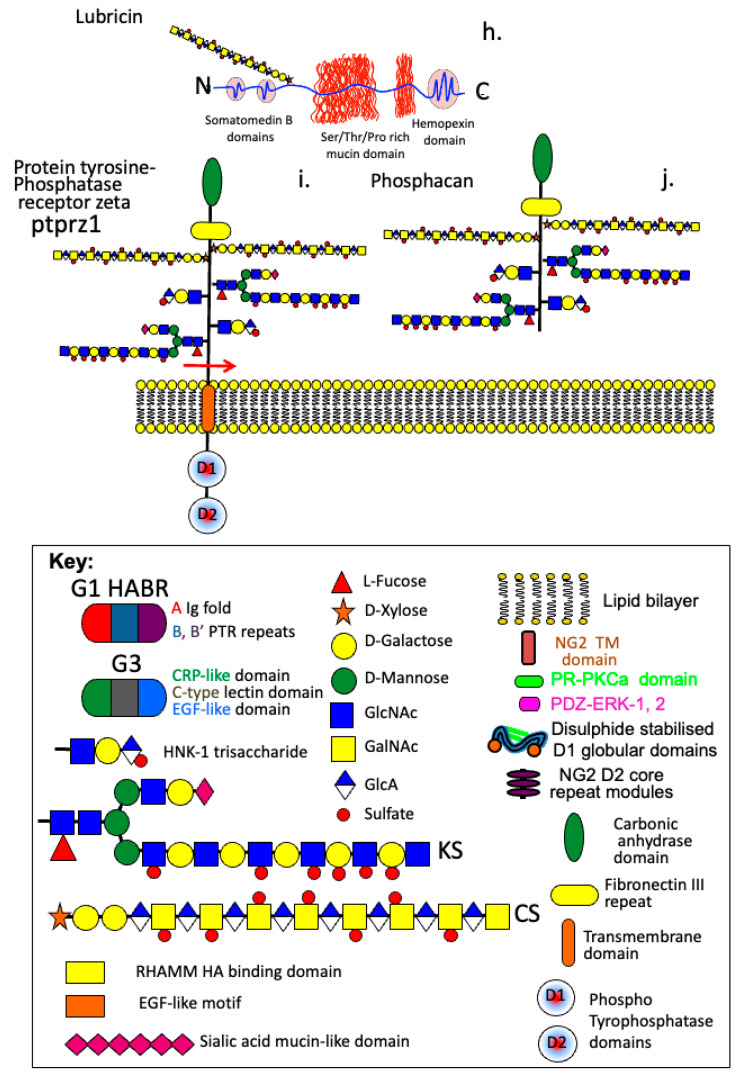
Schematics depicting the structure of neural aggrecan (CSPG1) (**a**), neurocan (CSPG3) (**b**), and brevican (CSPG7) (**c**). These are all members of the lectican proteoglycan family. Versican (CSPG2) also occurs as a large 370 kDa core protein V0 isoform with a molecular weight of ~1000 kDa in its full-length form (**d**). Versican also occurs as several alternatively spliced iso-forms; however, these have yet to be identified in ocular tissues. Neurocan occurs as a 220 kDa core protein that is processed into 130 and 150 kDa core protein forms by proteolytic activity. Brevican occurs as a 145 kDa full-length form and an 80 kDa N terminally truncated form. Neural/glial antigen 2, an NG2 proteoglycan, is a modular 300 kDa transmembrane protein synthesised by oligodendrocytes and pericytes (**e**). NG2 has cytoplasmic cell signalling domains and extracellular domains interactive with ECM components. Interphotoreceptor ECM PGs, IMPG1 (**f**) and IMPG2 (**g**), protect photoreceptors from oxidative stress. Lubricin is a small CS boundary lubricative proteoglycan found in the surface of the cornea and has protective and anti-inflammatory properties also (**h**). Protein tyrosine phosphatase receptor zeta is a cell membrane precursor CS–proteoglycan (**i**) which is cleaved (arrow) close to the cell membrane releasing its ectodomain, phosphacan (**j**). These PGs are substituted with KS side and HNK-1 trisaccharide, increasing their ligand interactivities.

**Figure 8:**
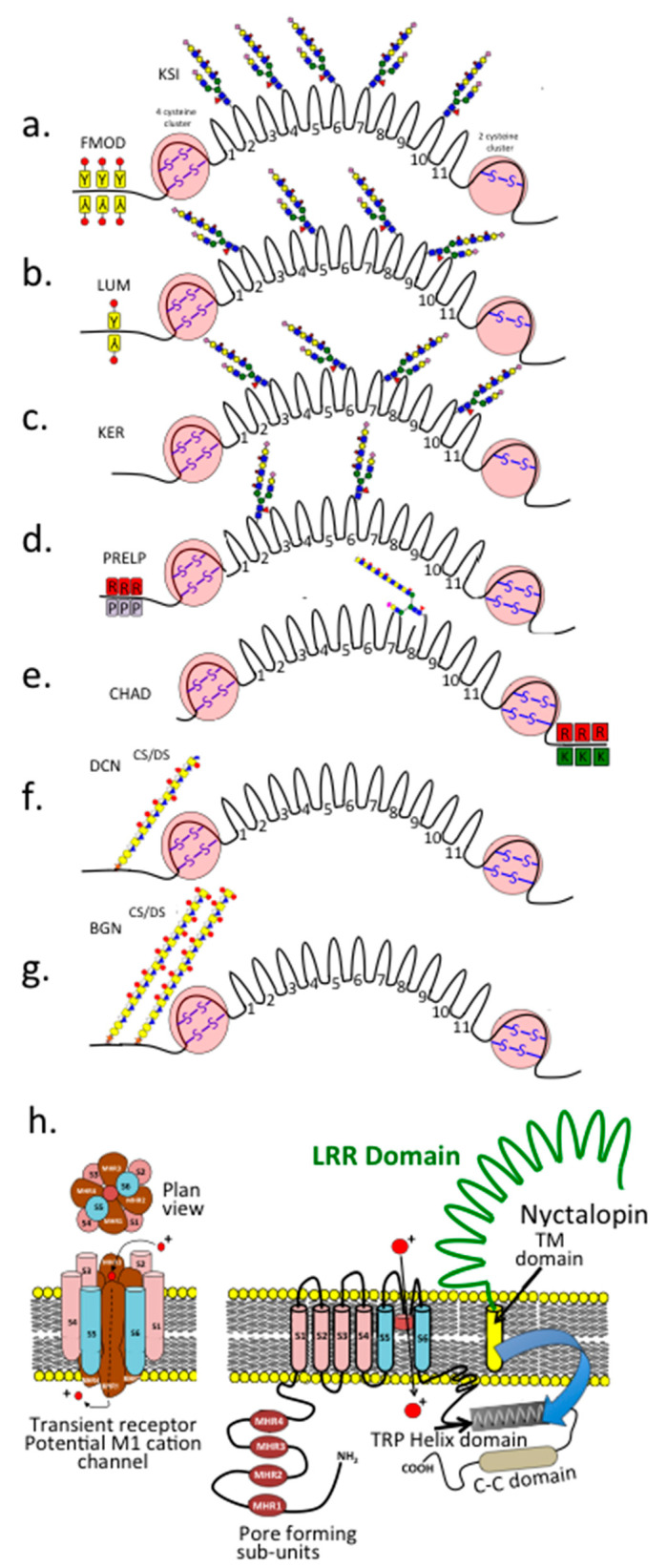
Schematic depiction of the boomerang type structural organisation of the SLRPs with their central leucine-rich repeat (LRR) domains, N and C disulphide stabilised globular domains, and KS and CS/DS substitution sites. Fibromodulin (FMOD) (**a**), lumican (LUM) (**b**), keratocan (KER) (**c**), PRELP/Prolargin (proline- and arginine-rich end leucine-rich repeat protein) (**d**), and CHAD (chondroadherin) (**e**) are KS substituted SLRPs. Decorin (DCN) (**f**) and biglycan (BGN) (**g**) are CS/DS SLRPs. FMOD and LUM possess several sulfated tyrosine residues in their N-termini, conferring HS-like properties (**a**,**b**). PRELP’s N-terminus, rich in Pro and Arg residues, binds strongly to heparin and HS, whereas its GAG components interact only weakly with ECM components. CHAD has a C-terminal Arg-Lys peptide module that interacts with heparin, HS, and cell-surface HSPGs (**e**). Nyctalopin (NYX) is a class II SLRP component of the bipolar ribbon synapse, which relays signals from photoreceptors to the retinal neural network for transfer to the ganglionic neurons and then to the brain via the optic nerve, essential in visual perception (**f**). NYX interacts directly with the G-protein coupled with the transient receptor potential melastatin cation channel M1 (TRPM1) and mGluR6 cell signalling pathway essential for the function of the ribbon synapse. This is depicted in the schematic in (**h**).

**Figure 9:**
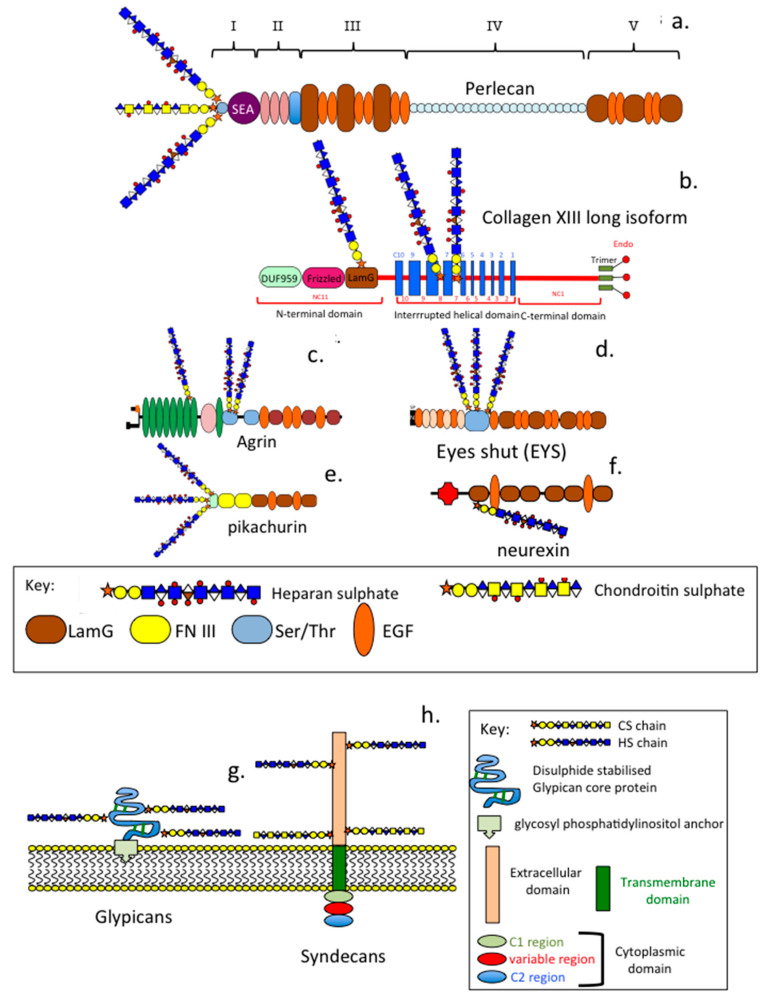
Schematic depictions of the structural organisation of HSPGs that have been identified in a range of ocular tissues. Multifunctional modular perlecan (**a**), collagen XVIII (**b**), and agrin (**c**). Specialised synaptic HSPGs such as eyes-shut (**d**), Pikachurin (**e**), and neurexin-α (**f**). Cell-surface PGs of the glypican (GPC1-6) (**g**) and syndecan families (SDC1-4) (**h**) are also shown as generic structures, as some members of these families contain additional GAG side chains.

Ocular PGs have diverse structural forms which reflects their variable functional properties in specific regions of the eye (Figure 7, Figure 8 and Figure 9).

LamG and EGF modules have important interactive properties with dystrophin-dystroglycan complexes which regulate cell–ECM communication. Perlecan, collagen XVIII, and agrin are basement membrane PGs and are highly interactive with a large range of ECM proteins, which aid in tissue stabilisation and sequester growth factors, as well as influence cell proliferation and differentiation aiding in tissue development and ECM remodelling in tissue responses. Pikachurin, eyes-shut, and the neurexin family stabilise and provide specificity to synaptic interactions and plasticity. Pikachurin interacts with dystrophin–dystroglycan complexes in photoreceptors as well as mGluR6, TRPM1 cation channels and GPR179 orphan receptors in bipolar neurons to stabilise the ribbon synapse, facilitating phototransductive and neurotransductive processes essential for visual functions.

### 8.1. The Lectican PGs

Aggrecan (Acan) is a 250 kDa core protein with KS and CS chains, featuring three globular domains (G1–G3) [131,132,133]. The G1 domain binds HA, forming aggregates that hydrate and fill space in tissues. In the brain, some CS chains are replaced by HNK-1 trisaccharide, increasing ligand interactivity during neural development [134]. Aggrecan plays a crucial role in cardiovascular [133] and neural development [135], and is extensively distributed throughout the retina [46] and sclera [12].

Aggrecan and versican (Vcan) share homologous N- and C-terminal domains [136,137]. Their G1 domains bind HA, while the G3 domains interact with ECM proteins, forming structures that store growth factors like TGF-β and BMPs. The G3 domain also contains EGF-like motifs and is subject to alternative splicing, enabling interactions with proteins such as COMP, fibulins, and tenascins, which are crucial for matrix organisation and tissue stability [136,138,139]. The C-type lectin motif in G3 can activate complement pathways [140]. Versican interactions with ECM proteins and cell-surface receptors, influences cell migration and proliferation. Versican occurs as splice variants (V0, V1, V2, V3, V4), with V0 being the largest (~1000 kDa). The central domain of versican contains GAG-α and GAG-β regions, with isoforms differing in GAG content. A bioactive G1 domain fragment, versikine, has roles in cell signalling and tissue remodelling in disease [141,142,143].

#### HNK-1 Content of Brain Aggrecan

The HNK-1 glycan motif on brain aggrecan plays important roles in neural crest development, synaptic plasticity, learning, and memory [50,132,134,144]. Its expression is tightly regulated in the nervous system. HNK-1 is also found on glycolipids, glycoproteins, and tenascin-R [145], where it influences neural stem cell activity and stabilises the GluR2, a glutamate receptor subunit [146]. As a component of perineuronal nets (PNNs), HNK-1 supports neural plasticity [50]; a deficiency of this can lead to impaired synaptic plasticity and spatial learning [147].

### 8.2. NG2 Proteoglycan

NG2 (CSPG4) is a type-1 transmembrane CSPG expressed by oligodendrocyte progenitor cells and pericytes in the retina [49,148,149]. It regulates intracellular signalling, cell migration, and cytoskeletal interactions. NG2’s extracellular domain can modulate neuronal networks and is cleaved into fragments in brain trauma with distinct functions. Abnormal behaviour of NG2 glial cells contributes to neurodegenerative diseases [148,150,151]. NG2 binds PDGF-AA, enhancing PDGFRα signalling, and ADAMTS4-mediated cleavage of NG2 promotes oligodendrocyte differentiation and re-myelination.

### 8.3. SPACRCAN (IMPG2) in the Eye

SPACRCAN (IMPG2), a 400 kDa CSPG in the interphotoreceptor matrix of the eye [48] contains six-sulfated CS chains and numerous oligosaccharides, constituting ~60% of its mass. SPACRCAN has RHAMM-HA binding domains, and HA-SPACRCAN aggregates organise the ECM and protect photoreceptors from oxidative stress through ROS [152].

### 8.4. Lubricin

Lubricin (PRG4) is a multifunctional proteoglycan, acting as both a boundary lubricant and an anti-inflammatory agent [153,154]. It occurs as proteoglycan and glycoprotein forms [155], containing somatomedin B, CS, KS, sialic acid-rich mucin, and a hemopexin domains. Lubricin’s mucin-rich central region is highly glycosylated, aiding in lubrication and heme detoxification via the hemopexin domain [156]. On the ocular surface, lubricin reduces inflammation by downregulating TNFα-stimulated NFκB activity and modulating Toll-like receptor signalling via CD44 [157]. It is also produced by mesenchymal stem cells and may support tissue homeostasis and regeneration in the CNS [158].

### 8.5. Phosphacan

Phosphacan, the extracellular domain of RPTP-zeta, is a neural proteoglycan with several isoforms (full-length, soluble, truncated) and can carry CS, KS, and HNK-1 modifications [159]. It is widely expressed in the CNS and retina, regulating neural development, cell adhesion, migration, myelination, and cognitive functions [160]. Phosphacan binds various ligands, including pleiotrophin, midkine, tenascin, NCAM, Ng-CAM, and contactin [161].

### 8.6. SLRPs

SLRPs regulate cell metabolism and tissue stability by interacting with cytokines, growth factors, and structural proteins [162,163]. FMOD’s sulfated tyrosines increase collagen binding [164]. LUM features modules that not only prevent melanoma migration but also support corneal repair [165,166,167,168,169,170,171]. The C-terminal Arg-Lys peptide module of CHAD modulates cellular activity by selectively binding to HS chains [172]. PRELP anchors perlecan [173] and other ECM components in basement membranes [174] while suppressing NF-kappaB activity in osteoclasts [175]. Nyctalopin (NYX) is essential for phototransduction, forming complexes with TRPM1 and mGluR6 to facilitate retinal signalling [175].

## 9. Diverse Functions of HSPGs in Ocular Tissues

HSPGs are found attached to cell surfaces (syndecans, glypicans) and in the ECM (perlecan, agrin, collagen XVIII) [176,177], while serglycin as the only known intracellular HSPG [178].

### 9.1. Perlecan

Perlecan is a large, modular proteoglycan that stabilises the extracellular matrix [179], mediates cell–ECM communication [180], sequesters growth factors [181], and participates in cell signalling [182]. Domain I contains HS and in some cases CS, and sequesters growth factors, while domain II interacts with lipids relevant for retinal pigment epithelium (RPE) lipid processing [183,184]. Perlecan helps regulate lipid metabolism and inhibits complement activation in BrM [104,185], protecting against AMD [186]. Ageing reduces HS chains on perlecan, weakening this protection and increasing AMD risk. 

### 9.2. Collagen XVIII

Collagen XVIII, a modular HSPG with anti-angiogenic properties (endostatin domain), is present in most basement membranes, including the RPE, BrM, choroid, and sclera [7,187]. It is essential for ocular matrix stability and vision. Mutations can cause severe retinal disorders, such as Knobloch syndrome, and its endostatin domain may help control abnormal blood vessel growth in AMD [112].

### 9.3. Agrin

Agrin is a 400 kDa HSPG involved in neural development, synaptic stability, and tissue repair [188,189,190,191]. It interacts with LRP4 and α-dystroglycan, and can exist as multiple isoforms, including HS/CS hybrids. Agrin is also important for blood–brain barrier integrity and promotes regeneration in injured tissues [118,192].

### 9.4. Synaptic HSPGs

Specialised HSPGs such as pikachurin [62,122], eyes-shut [193], and neurexins [65,66,67,194] are crucial for synaptic stability and specificity in neuronal tissues. Their interactive HS chains regulate cellular activity and tissue organisation by binding growth factors and ECM proteins [108,110], highlighting the broad functional diversity of HSPGs in the eye [195].

## 10. Cell-Surface HSPGs

### 10.1. Syndecans

Syndecans (SDC 1–4) are cell-surface heparan sulfate PGs found in the CNS and ocular tissues. N-syndecan is highly expressed in neural tissues, especially during retinal development [196], and may help form the retinal neural network [197]. Syndecans promote axon regeneration, enhance HS chain sulfation (notably SDC-2), and support neovascularisation. They act as co-receptors that interact with ECM components, regulate cell behaviour [198,199], organise ECM [200,201], promote cell adhesion, and participate in cell signalling. Protease activity releases syndecan ectodomains, which can act as soluble antagonists or competitive inhibitors [202]. Matrix metalloproteinases (MMPs) modulate syndecan bioactivity by cleaving ectodomains [203].

### 10.2. Glypicans

Glypicans (GPC 1–6) are cell-surface PGs in the nervous system, attached via a glycosylphosphatidylinositol anchor [204]. Unlike syndecans, glypicans lack cytoplasmic domains but modulate cell signalling through ECM interactions [205]. The core protein is lipid-modified for membrane anchoring, and Notum deacylase releases glypicans from the cell surface [206]. Notum also inactivates Wnt signalling, affecting cell proliferation and differentiation. Glypicans regulate HH, Wnt, BMP, and FGF signalling, either stimulating or inhibiting these pathways [205,207,208]. GPC3 inhibits HH signalling [207], while GPC5 stimulates it [209]. Metalloprotease ADAM17 cleaves GPC-1, releasing soluble ectodomains that can antagonise cell-bound glypicans, implicating MMPs in glypican bioactivity regulation [210].

## 11. Conclusions

This review highlights the essential roles of ocular PGs in building, stabilising, and protecting eye tissues, which are crucial for vision. As key effector proteins, they contribute to the eye’s complexity and function. Future therapies targeting these proteins may improve treatment for ocular diseases. According to the World Health Authority, 285 million people worldwide have visual impairment, including 39 million who are blind and 246 million with low vision. Notably, 80% of visual impairment is preventable, emphasising the urgent need for better preventative and therapeutic strategies as the global population ages.

## Data Availability

No new data were created or analyzed in this study. Data sharing is not applicable to this article.
